# Conservation genomics of an endangered arboreal mammal following the 2019–2020 Australian megafire

**DOI:** 10.1038/s41598-023-27587-3

**Published:** 2023-01-10

**Authors:** Monica L. Knipler, Ana Gracanin, Katarina M. Mikac

**Affiliations:** Centre for Sustainable Ecosystem Solutions, School of Earth, Atmospheric and Life Sciences, Faculty of Science, Medicine and Health, University of Wollongong, Wollongong, Australia

**Keywords:** Population genetics, Conservation biology

## Abstract

The impacts of a changing climate threaten species, populations and ecosystems. Despite these significant and large-scale impacts on threatened species, many remain understudied and have little to no genetic information available. The greater glider, *Petauroides volans*, is an endangered species highly sensitive to the predicted changes in temperature under a changing climate and was recently severely impacted by a megafire natural disaster (85% estimated population loss). Baseline genetic data is essential for conservation management and for detecting detrimental changes in fire-effected populations. We collected genetic samples within 2 years post the 2019–2020 catastrophic Australian bushfires to examine adaptive potential, baseline genetic diversity and population structure, across their southern range in the state of New South Wales. Population genomic analyses were conducted using 8493 genome-wide SNPs for 86 greater glider individuals across 14 geographic locations. Substantial genetic structure was detected across locations, with low genetic diversity and effective population sizes observed in isolated areas. Additionally, we found signals of putative adaptation in response to temperature in greater gliders using a genotype-environment association analysis. These findings have important implications for the management of greater glider populations by identifying at-risk populations and identifying adaptive potential. We demonstrate the importance of baseline genetic information for endangered species as a practical approach to conservation. This is particularly important given the threat that changes in temperatures and megafire events, as predicted under a changing climate, poses for this species.

## Introduction

When natural disasters occur at large scales, such as tsunamis, earthquakes, hurricanes, or wildfires, they can have profound effects on a species not only in the short term^1,2^ but can also have long lasting and ongoing effects^3,4^. Natural disasters are likely to also be exacerbated by climate change^5,6^, and their impact can be severe, as a single event can result in the sudden loss of millions of animals from populations across their range^1,2^. Populations that suddenly experience significant reductions in effective population size can result in a population bottleneck^7^. Changes to these populations include a loss of genetic diversity^8,9^, compromised ability to adapt to environment changes^10^, and an increase in extinction likelihood thorough various genetic and demographic processes^11–14^. As a result, conserving genetic diversity within a species population is critical to ensure resilience and evolutionary potential in response to anthropogenic climate change^15,16^.

Conservation genetics as a management tool is becoming increasingly important, particularly for small populations and threatened species^17,18^. Genetic information can identify management units for conservation, assess population size, quantify connectivity, detect hybridisation, and evaluate the potential for populations to adapt to environmental changes^16,17,19,20^. Genetic diversity, contemporary effective population size and adaptive potential are vital pieces of information that can aid in the conservation of threatened species, as it tells of a populations ability to persist and adapt to changes in their environment^21–24^. Despite the importance of these measurements, many threatened species have missing or very limited baseline genetic data that can be used to assess the conservation status of the species, inform the direct management of wild populations, or even delineate their range^25–27^.

The greater glider (*Petauroides volans*) is a nocturnal, canopy-dwelling, gliding marsupial found along the east coast of Australia^25,28^. Greater gliders are highly dependent on eucalypt forests, as they have specialist eucalypt diets^29–31^ and are reliant on large hollows for sheltering and raising offspring^28,32,33^. The species has experienced significant decline as a result of habitat loss^34,35^, wildfire^36,37^ and the effects of climate change^38–40^. Over the past 20 years, long-term studies have identified rapid declines in populations^38,41–43^ as well as local extinction, such as at Booderee, New South Wales (NSW)^42^. Consequently, the species has recently been uplisted from vulnerable to endangered under the Australian *Environment Protection and Biodiversity Conservation Act 1999* (EPBC Act) and is listed as vulnerable under the International Union for Conservation of Nature (IUCN) Red List of Threatened Species^44^.

Increases in annual temperatures and frequency of heatwaves has been shown to have an over-riding, and devastating, impact on greater glider occupancy throughout its range^38,40^. Unlike many other mammals, the greater glider becomes hyperthermic at temperatures above 20 °C^45^, making them particularly sensitive to ambient temperature changes and changes to the availability of water^40^. In one study, it was found that over the past four decades, an increase in hot nights (> 50 occasions per decade) was associated with a decline (63% to 10%) in the total area of suitable climatic conditions^40^. This indicates that significant range contraction to cooler and wetter habitat at high elevations is likely to occur^38^. Furthermore, heatwave events are also strongly associated with a greater probability for catastrophic wildfires, and the Australian megafire event of 2019–2020 is likely to occur again^46^. Greater gliders experienced an immediate population loss of an estimated 85% following the 2019–2020 bushfires^47^. These events pose a serious threat for greater glider populations unless they can adapt to changing environmental conditions^48,49^. However, the absence of baseline population genetic data hinders any opportunity for informing practical management actions^50–52^.

**Figure 1:**
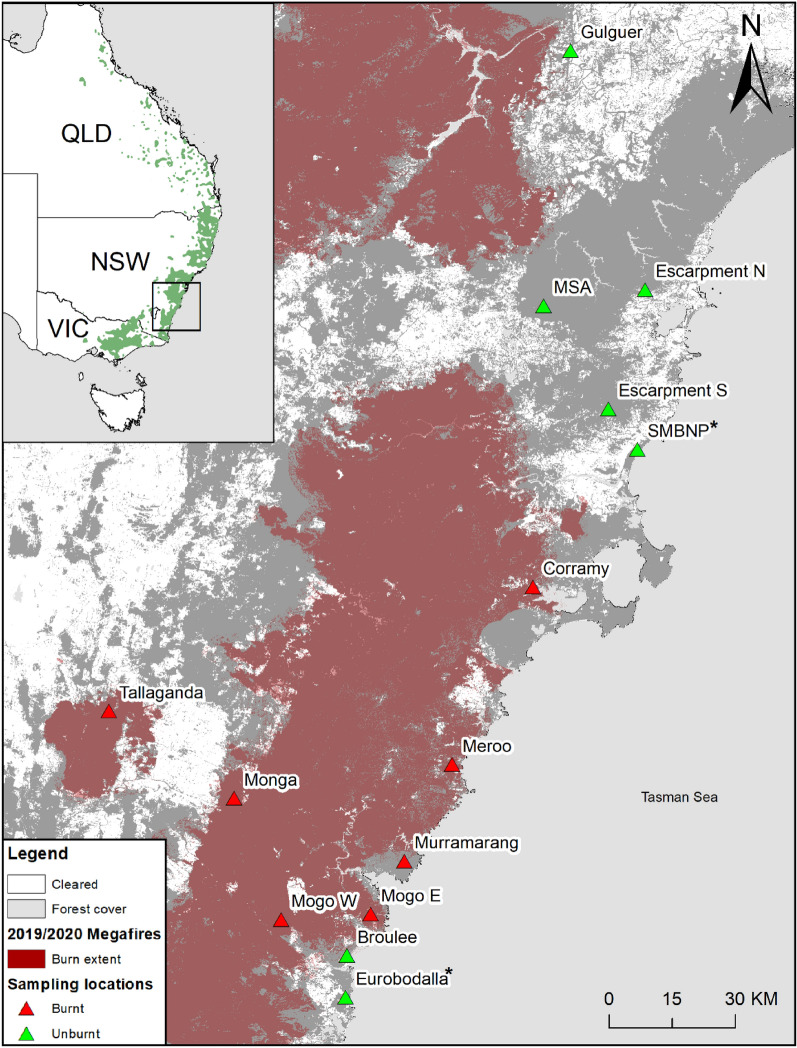
Location of the 14 study areas in southern NSW, Australia. Red shading indicates areas burnt during the 2019–2020 megafires^97^. Sites indicated with * are locations of two previously state listed endangered populations. Forest cover layer is from^113^. Top left corner: the green shading represents greater glider habitat as mapped by the Australian Government (Species of National Environmental Significance spatial database) from^114^. The distribution spans across the Australian states of Victoria (VIC), New South Wales (NSW) and Queensland (QLD). This map was generated using ArcGIS 10.7.1^115^.

Baseline genetic data collected before populations decline offer the opportunity to inform conservation management and reduce extinction risk^16,26^. For the endangered greater glider, there is no genome-wide single nucleotide polymorphism (SNP) data available across its entire range in the state of NSW (Fig. 1). The species is notoriously difficult to capture for genetic studies (they are obligate canopy dwellers) and only recently has the greater glider been classified as three separate species^25^. However, these three species were identified using genetic samples taken from Victoria and Queensland, with a significant gap occurring in NSW (53% of the total distribution of greater gliders). Genetic analysis has been performed at smaller scales in NSW, investigating greater glider metapopulation structure in only one fragmented forest, using microsatellite data^53–55^. Under NSW state legislation, the species was uplisted to endangered in 2022, and three small populations were listed as endangered under the NSW *Biodiversity Conservation Act 2016.* For these endangered populations, baseline genetic research is a priority due to their isolation and vulnerability to stochastic changes^56–58^.

Our aim was to provide crucial baseline genetic data for greater gliders before genetic impacts from the 2019–2020 catastrophic bushfires could be observed. Greater gliders have a generation time of 7–8 years^59,60^, and this study was conducted within 2 years of the 2019–2020 Australian bushfires. This meant that the adults captured in this study were likely conceived pre megafire. It is possible that populations were already experiencing genetic declines because of habitat fragmentation and isolation, thus the ongoing impacts of the megafire is likely to compound these effects. Baseline genetic data is essential for greater glider conservation and management, as it will allow researchers to monitor changes in the effective population size and genetic diversity of fire-effected genetic clusters, and act accordingly.

## Methods

### Study area

Our study was undertaken in the southern half of New South Wales, spanning an area of approximately 15,000 km^2^ (Fig. 1). Fourteen locations were sampled across 2020–2021 that represent a range in elevation from coastal areas (8–170 m.s.l.) to higher elevations (415–1179 m.s.l.). We collected greater glider DNA from sites that were affected by the 2019–2020 bushfires (Corramy, Meroo, Murramarrang, Mogo E, Mogo W, Monga and Tallaganda), unburnt endangered populations (SMBNP and Eurobodalla), and unburnt sites (Gulguer, MSA, Escarpment N, Escarpment S, Broulee) (Fig. 1). Though gliders were caught from an area unburnt in Murramarrang, the immediate surrounding forests were burnt thus having substantial impact on the population within this region.

### Sample collection

Tissue was sampled from 85 wild greater gliders from 14 locations. An additional five samples were obtained from donations made by the public (i.e., dead specimens found in situ) (Supplementary 1). Two methods of live capture were utilised in this study. The first was the climb and catch method^61^. This involved tree climbing to capture a glider from its hollow. To identify a glider residing within a hollow, stag-watching and a tracking method were undertaken^61^. The second method was the branch-shake method^61–63^. This involved spotlighting to detect greater glider eyeshine. If the glider was found to occur at the far end of a thin branch, and at the required height (< 12 m), a throw line was launched over the branch and was used to shake the branch. The glider would then glide to the ground, where it was slow and readily captured by hand.

All captured individuals were weighed, sexed and measured. We collected DNA samples from greater gliders using a 2 mm metal ear punch (Able Scientific, Australia). The metal ear punch was sprayed with 70% ethanol and flamed for sterilization. Once cooled, a clip was taken from the outside edge of the ear and stored in sterilized vials containing 80–95% ethanol. DNA was kept at − 20 °C prior to DNA extraction.

All research was performed in accordance with relevant guidelines and regulations. The experimental protocols were approved by a University of Wollongong Animal Ethics committee (AE19/02). Research was conducted under a NSW DPIE Scientific Licence (SL101968).

### DNA extraction and genomic sequencing

A total of 94 greater glider samples (2 mm ear tissue from 90 individuals, with four duplicates) were plated and sent to Diversity Arrays Technology Pty Ltd (DArT) (Australia), for DNA extraction and next generation sequencing.

For DNA extraction DArT removed the ethanol and incubated the tissue samples with T1 Buffer and proteinase K at 60 °C overnight. The lysates were aspirated to a new plate and the DNA was bound to NucleoMag B-beads before being washed and eluted with a Tecan 100 robot and DArT PL script (maximum concentration 50–100 ng/µl). To find the best genome complexity reduction method for sequencing, DArT tested and optimized different restriction enzyme combinations. They selected the PstI-SphI enzyme combination as the optimal method for the *Petauroides* genus. DNA digestion and ligation followed the steps outlined in Kilian et al. ^64^, except two different kinds of adaptors were used for compatibility with the PstI and SphI overhangs.

DNA fragments were amplified using Polymerase Chain Reaction (PCR) and the following thermal cycling conditions: initial denaturation step for 1 min at 94 °C, 30 cycles of (94 °C for 20 s, 58 °C for 30 s and 72 °C for 45 s) and a final elongation step at 72 °C for 7 min. Amplified DNA samples were pooled and underwent Illumina c-Bot bridge PCR. The library then underwent 77 sequencing cycles on an Illumina Hiseq2500, providing 2,500,000 sequences per barcode per sample. SNPs were called using DArT analytical pipelines (DArTsoft14), and an average read depth of 20 reads per locus ensured calling quality. Additional quality control procedures can be found in Kilian et al. ^64^. Genomic regions around SNPs were aligned to the closest relative available: the Leadbeaters possum (*Gymnobelideus leadbeateri,* McCoy) reference genome ‘GCA_011680675.1 LBP_v1’ using an E-value of 5e−7 and a minimum sequence identity of 70%. SNP markers were scored “0”, “1” and “2” representing reference allele homozygote, SNP allele homozygote and heterozygote respectively.

### Filtering of greater glider genome-wide SNPs

We stringently filtered the genome-wide SNPs from DArT using the *dartR* v 2.0.4 package^65^ in R 4.0.2^66^. Loci were removed if (1) the average repeatability of alleles at a locus was < 0.99, (2) the call rate of loci was < 0.80, (3) minor allele frequency for a locus was < 0.01, (4) read depth was < 5 or > 100, and (5) if they were secondary SNPs with the possibility of being linked. It was ensured no monomorphic loci remained in the dataset.

### Outlier detection

Outlier loci (potential loci under selection) were detected using a differentiation-based analysis and an environmental association analysis. The differentiation-based analysis was conducted using the *pcadapt* v 4.3.2 package in R^67,68^. Pcadapt uses principal components to detect candidate loci under selection^67,68^. The first two principal components were retained (K = 2) based on the inflection point of the screeplot and the proportion of explained genetic variation from K = 1 to 20 (Supplementary 2). A list of outlier loci was generated using the *qvalue* v 2.20.0 package^69^, and SNPs were considered outliers when their qvalue was < 0.10 (False Discovery Rate expecting 10% of the outlier loci to be false positives).

A Redundancy Analysis (RDA) was conducted using the *vegan* v 2.5-7 package in R to detect outlier loci that may be associated with environmental variables^70^. This multivariate analysis has proved effective in identifying environmental variables potentially responsible for selection in many species^71–73^, including a potential temperature adaptation in another Australian folivore, the koala (*Phascolarctos cinereus,* Goldfuss)^74^.

RDA climate variables were obtained for each greater glider sampling location. These values were taken from the WorldClim database using a spatial resolution of 30 s and the *raster* v 3.6 package in R^75^. Environmental variables were filtered to avoid high correlations (Pearson correlation coefficient > 0.6) while prioritizing variables of interest based on the knowledge that greater gliders are sensitive to heat and water availability^40,45^. As a result, three environmental variables were retained for the RDA: BIO5 (maximum temperature of the warmest month), BIO10 (mean temperature of the warmest quarter), and BIO16 (precipitation of the wettest quarter).

As RDA requires a complete genetic data frame, the ‘snmf’ function in *LEA* v 3.0.0 was used to impute missing data after testing 10 runs of K 1 to 10 (best K = 3 and run = 10). After running the RDA, the significance of each constrained axis was calculated using the “anova.cca” function in *vegan*. Outlier SNPs were identified on the first three RDA axes. When a SNPs loading was ± 3 standard deviation from the mean, it was flagged as an outlier (candidate adaptive loci)^72^, and these were assigned to either BIO5, BIO10 or BIO16 based on the strongest correlation^72^. The three environmental variables had a Variance Inflation Factor (VIF) less than five, so multicollinearity was not an issue (VIF range 1.09–1.56).

Pcadapt and RDA outlier loci were removed from the dataset to ensure neutral SNPs were used for the following population genomic analyses.

### Greater glider genetic diversity calculations

Greater glider population genetic diversity was measured in the form of observed and expected heterozygosity of SNPs. Average observed heterozygosity (Ho), expected heterozygosity (Hs) and the inbreeding coefficient (Fis = 1 − Ho/Hs) were calculated for each locus and greater glider sampling locations using the R package *hierfstat* v 0.5-10^76^ and equations from Nei^77^.

### Detecting greater glider genetic structure

#### Pairwise FST calculation

Genetic distances of sampling sites (putative ‘populations’) can be quantified with a pairwise F_ST_ matrix, where values are produced for each population comparison. Values near 0 indicate no genetic differentiation between populations, while higher values indicate genetic differentiation and structure (maximum possible value is < 1 and is dependent on the expected heterozygosity within-population)^78,79^.

The *StAMPP* v 1.6.3 R package generates F_ST_ values that are unbiased by sample size^80,81^. Greater glider pairwise F_ST_ values were generated using *StAMPP* and 10,000 bootstraps across loci to generate p-values. A Bonferroni correction for multiple testing generated a new p-value of 0.0005 to infer significance^82^.

#### DAPC and STRUCTURE analyses

Geneticists use programs such as STRUCTURE and Discriminant Analysis of Principal Components (DAPC) to group individuals with similar genetic patterns into genetic clusters (*K*). STRUCTURE detects genetic clusters (*K*) with a Bayesian iterative clustering algorithm^83^, while DAPC uses a multivariate method to assign individuals to genetic clusters with sequential *K*-means and model selection^84^. We decided to report the results of both programs here since they use different techniques and have their own limitations^85–87^.

We ran STRUCTURE v 2.3.4 using 8 replicates of *K* 1 to 15^83,88^. The running length was 10,000 for the burn-in period and 10,000 for the MCMC replications. An admixture model was selected, and allele frequencies were selected as “correlated among populations”. All other parameters were kept default. Results were uploaded to Structure Harvester (Web v 0.6.94)^89^ where the optimal *K* was selected after analysing ∆*K*, the STRUCTURE plots and the posterior probability^90^.

Greater glider DAPC genetic clusters were determined de novo using the ‘find.clusters’ function in *adegenet* v 2.1.5^91^ and a maximum *K* value of 15. The optimal number of clusters was chosen based on the lowest BIC value (Supplementary 3)^86^. The number of principal component axes (PCA’s) retained in the DAPC analysis was determined through the ‘xvalDAPC’ function (maximum PC’s = 100).

#### Analysis of molecular variance

An Analysis of Molecular Variance (AMOVA) calculates the level of genetic differentiation within samples, between samples within populations, and between populations. We conducted an AMOVA in *poppr* v 2.9.3 using 9999 permutations and the geographic locations as putative populations. We examined the proportion of genetic differentiation that was apportioned between greater glider sampling locations, between greater gliders within sampling locations, and between greater glider samples themselves. A second and third AMOVA was conducted after reassigning greater glider individuals to the de novo groupings detected with STRUCTURE and the DAPC analysis, to see if this explained more of the genetic variation.

#### Pearson principal component analysis

Principal Component Analyses are multivariate analyses that are used to plot multi-dimensional population substructure. We ran a Pearson Principal Component Analysis (PCA) to visualise the genetic structure of NSW greater gliders using the ‘gl.pcoa’ function in *dartR*, and individuals were plotted on the first four axes. The 14 sampling sites were colour coded so that patterns of genetic variation could be observed.

### Greater glider isolation by distance analysis

Once genetic structure was identified in NSW greater gliders, it was tested to see if some of the genetic structure could be explained by geographic distance. To do this, an Isolation By Distance (IBD) analysis tested whether the geographic distances between greater glider sampling sites correlated with the genetic distances of the NSW sites. An IBD analysis was generated for the greater glider dataset using the “gl.ibd” function in *dartR,* the “mantel” function in *vegan* and 9999 permutations. The log of the Euclidean distances were compared to the genetic distances of populations (pairwise F_ST_/1 − F_ST_).

### Effective population size estimates

Small effective population sizes can decrease genetic diversity, increase inbreeding and put a population at risk of extinction through genetic drift^92^. NeEstimator uses a single-sample linkage disequilibrium method to calculate contemporary effective population sizes (Ne) (i.e. the effective population size for the time the samples were obtained)^93^. Ne values were calculated for greater glider sampling sites with more than five individuals, using the *RLDNe* v 0.1.0 package^93^ and an allele frequency cutoff of 0.05. A random mating system was selected, and all other default parameters were used. Upper and lower confidence intervals were generated with the jackknife resampling method as it performs better than the parametric method^94^.

## Results

### Genetic markers

#### DArTseq results

DArTseq returned 18,807 genome-wide SNPs from 90 greater glider tissue samples (Supplementary material 1). Three samples failed the DArTseq quality control stage: one sample that was collected from a captured greater glider (GG-BWH-20, location: Escarpment S), and two samples that were collected from a greater glider body that was euthanized and donated to us by Ulladulla Veterinary Clinic after it was brought in by a member of the public (GG-D-04, location: Kings Point).

#### Filtering of greater glider samples and genome-wide SNPs

After receiving the DArTseq results, we filtered out additional samples from the dataset. We excluded two samples due to incorrect species identification and microbial contamination from Mogo and Shallow Crossing populations. Lastly, three duplicate samples were excluded. Locus metrics were recalculated after the individuals were removed, using the ‘gl.recalc.metrics’ function in *dartR*. Once additional locus metrics were filtered as per the methods section, 8623 genome-wide SNPs and 86 greater glider individuals remained from 14 geographic locations (Fig. 1, Table 1).

**Table 1:** Number of greater glider samples (*n*) collected from the 14 geographic locations.

Sampling site	*n*	Ho (± SD)	Hs (± SD)	Fis (± SD)
Gulguer	1	0.163 (0.370)	–	–
MSA	9	0.206 (0.203)	0.243 (0.197)	0.116 (0.379)
Escarpment N	2	0.202 (0.332)	0.169 (0.249)	− 0.279 (0.524)
Escarpment S	2	0.167 (0.284)	0.215 (0.310)	0.059 (0.565)
SMBNP	15	0.175 (0.194)	0.188 (0.191)	0.056 (0.284)
Corramy	5	0.104 (0.210)	0.102 (0.184)	− 0.027 (0.396)
Meroo	5	0.111 (0.200)	0.116 (0.191)	0.017 (0.367)
Murramarang	6	0.108 (0.198)	0.108 (0.182)	− 0.008 (0.338)
Mogo E	2	0.105 (0.246)	0.111 (0.235)	− 0.068 (0.559)
Mogo W	1	0.105 (0.306)	–	–
Broulee	10	0.104 (0.181)	0.107 (0.174)	0.026 (0.297)
Eurobodalla	6	0.090 (0.190)	0.089 (0.172)	− 0.018 (0.347)
Monga	20	0.126 (0.163)	0.139 (0.168)	0.078 (0.267)
Tallaganda	2	0.189 (0.305)	0.213 (0.297)	− 0.037 (0.574)
Total	86	0.139	0.147	0.055

Observed heterozygosity (Ho), expected heterozygosity (Hs) and the inbreeding coefficient (Fis) is reported for each sampling site, along with the standard deviation (SD). These results were generated using 8493 genome-wide SNPs.

### Candidate loci under selection

**Figure 2:**
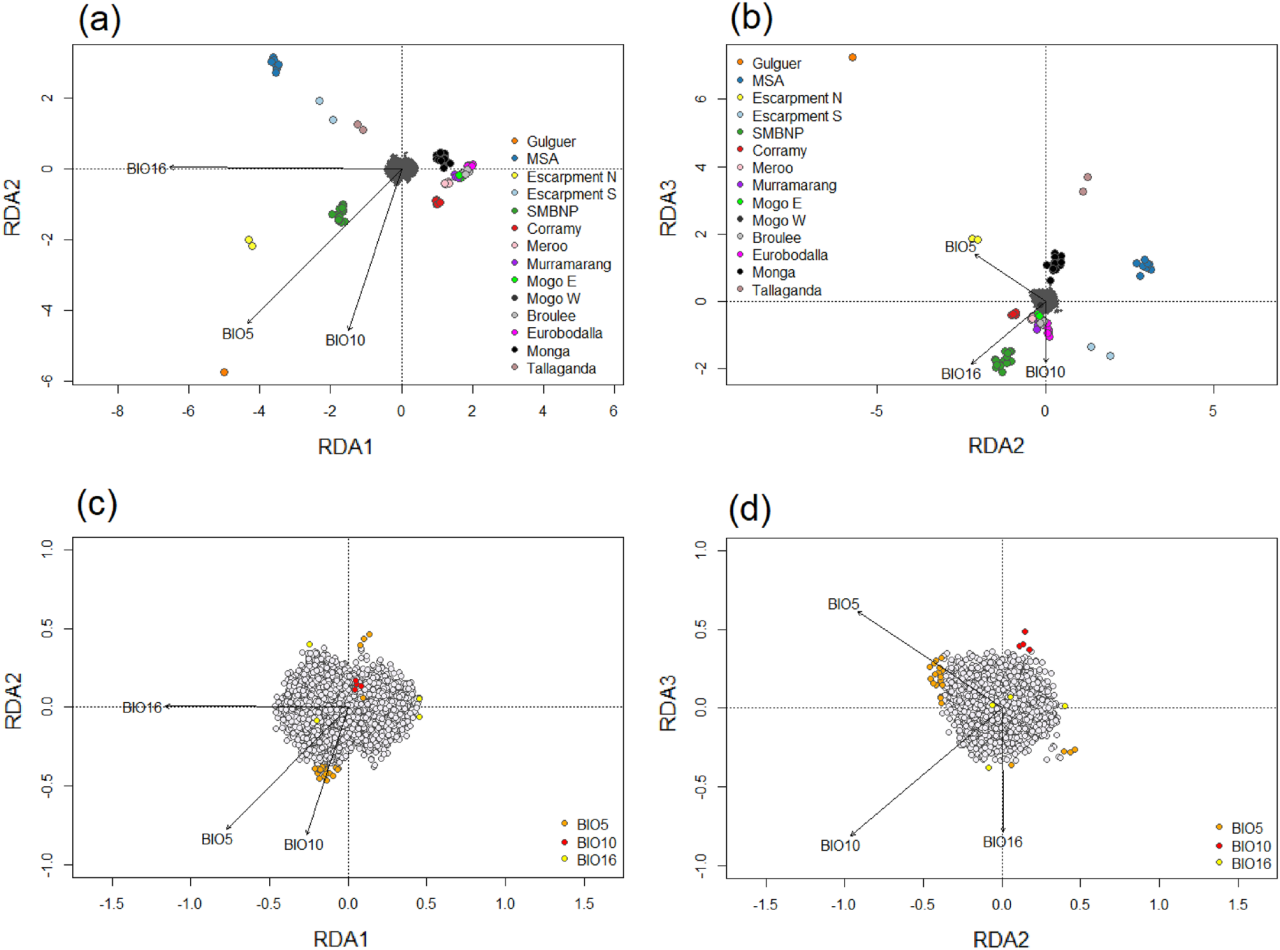
Plots show Redundancy Analysis (RDA) constrained axes 1 v 2 (**a**,**c**) and 2 v 3 (**b**,**d**). The pointed arrows represent environmental variables BIO5 (maximum temperature of the warmest month), BIO10 (mean temperature of the warmest quarter), and BIO16 (precipitation of the wettest quarter). (**a**,**b**) 8623 genome-wide SNPs are shown in grey. Each greater glider individual is coloured to represent their geographic location. (**c**,**d**): 8623 genome-wide SNPs are shown here, with neutral SNPs coloured grey. Putative adaptive loci are coloured orange when correlated with BIO5 (n = 66), red with BIO10 (n = 4) and yellow with BIO16 (n = 4).

The differentiation-based outlier detection method (pcadapt) detected 58 outlier loci potentially under selection in the greater glider genome, while the environmental-association analysis (RDA) detected 74 outlier loci as candidate adaptive loci (Fig. 2). Two common loci were detected by both programs, giving a combined total of 130 outlier loci.

Each of the three constrained RDA axes were significant (p = 0.001), and together they explained 14.0% of the genetic variation (adjusted R^2^ = 0.108). Of the 74 outlier loci detected by the RDA, 66 loci were associated with maximum temperature of the warmest month (BIO5), four were associated with mean temperature of the warmest quarter (BIO10), and four were associated with precipitation of the wettest quarter (BIO16) (Fig. 2c,d). The candidate adaptive loci were strongly associated with BIO16 on RDA axis 1 (Pearson correlation coefficient = − 0.66), and BIO5 and BIO10 on RDA axis 2 (− 0.65, − 0.68 respectively) (Fig. 2).

The outlier loci detected by pcadapt and RDA were removed prior to population genomic analyses, leaving a neutral dataset of 8493 genome-wide SNPs.

### Genetic diversity of greater glider populations

The observed heterozygosity of greater glider sampling sites ranged from 0.090 (Eurobodalla) to 0.206 (MSA) (Table 1). Observed heterozygosity was lower than expected at sampling site MSA, Escarpment S, SMBNP, Meroo, Mogo E, Broulee, Monga and Tallaganda. Observed heterozygosity was higher than expected at sampling site Escarpment N and Corramy. The inbreeding coefficients remained close to zero, however MSA had an excess of homozygotes (F_IS_ = 0.116) and Escarpment N had an excess of heterozygotes (F_IS_ = − 0.279) (Table 1). There were only two samples taken from Escarpment N however, so this value should be observed with caution.

### Spatial genetic structure of greater gliders

#### Pairwise F_ST_ results

There was significant genetic differentiation between greater glider sampling locations, with a high average F_ST_ value of 0.244 (± SD 0.166). Significant pairwise F_ST_ values ranged from low genetic differentiation between Gulguer and MSA (F_ST_ = 0.041) to extreme genetic differentiation between the greater glider at Gulguer and those at Eurobodalla (F_ST_ = 0.666) (Table 2). While the greater glider from Gulguer was genetically similar to those at MSA (F_ST_ = 0.041), it was significantly differentiated from all other geographic locations (Average F_ST_ = 0.474, ± SD = 0.174) (Table 2).

**Table 2:** Greater glider pairwise F_ST_ for 14 sampling locations (below the diagonal).

Sampling sites	Gulguer	MSA	Escarp-ment N	Escarp-ment S	SMBNP	Corramy	Meroo	Murra-marang	Mogo E	Mogo W	Broulee	Eurobodalla	Monga	Tallag-anda
Gulguer	–	0.000	0.000	0.000	0.000	0.000	0.000	0.000	0.000	0.000	0.000	0.000	0.000	0.000
MSA	0.041	–	0.000	0.000	0.000	0.000	0.000	0.000	0.000	0.000	0.000	0.000	0.000	0.000
Escarpment N	0.336	0.102	–	0.000	0.000	0.000	0.000	0.000	0.000	0.000	0.000	0.000	0.000	0.000
Escarpment S	0.200	0.084	0.247	–	0.000	0.000	0.000	0.000	0.000	0.7778*	0.000	0.000	0.000	0.000
SMBNP	0.318	0.164	0.280	0.081	–	0.000	0.000	0.000	0.000	0.000	0.000	0.000	0.000	0.000
Corramy	0.617	0.299	0.528	0.297	0.205	–	0.000	0.000	0.000	0.000	0.000	0.000	0.000	0.000
Meroo	0.573	0.278	0.491	0.243	0.178	0.171	–	0.000	0.000	0.000	0.000	0.000	0.000	0.000
Murramarang	0.602	0.307	0.519	0.280	0.196	0.194	0.085	–	0.000	0.000	0.000	0.000	0.000	0.000
Mogo E	0.583	0.226	0.478	0.173	0.146	0.206	0.083	0.103	–	0.1168*	0.000	0.000	0.0029*	0.000
Mogo W	–	0.153	0.446	–0.010	0.111	0.225	0.078	0.107	0.018	–	0.000	0.000	0.9992*	0.000
Broulee	0.611	0.340	0.534	0.306	0.221	0.209	0.114	0.118	0.046	0.061	–	0.000	0.000	0.000
Eurobodalla	0.666	0.342	0.575	0.356	0.241	0.271	0.174	0.182	0.143	0.186	0.134	–	0.000	0.000
Monga	0.502	0.297	0.435	0.205	0.173	0.126	0.052	0.058	0.012	–0.023	0.054	0.088	–	0.000
Tallaganda	0.207	0.085	0.266	0.090	0.169	0.357	0.302	0.337	0.228	0.087	0.349	0.392	0.247	–

The corresponding p-values are shown above the diagonal. Results were generated from 8493 genome-wide SNPs and 86 greater glider individuals.

*Non-significant p-value after Bonferroni correction for multiple testing.

#### STRUCTURE program results

**Figure 3:**
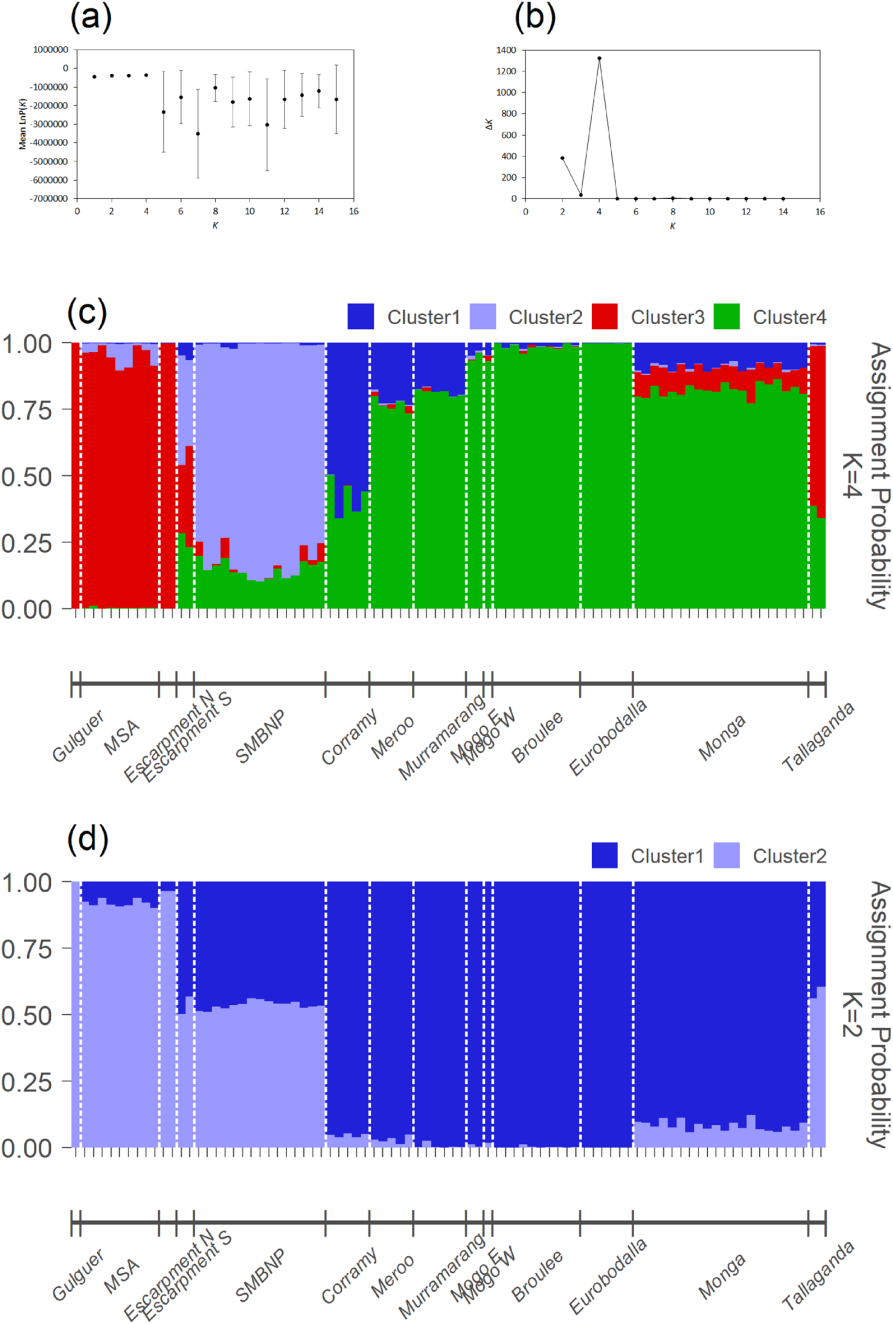
(**a**) Structure Harvester results displaying the mean likelihood of each genetic cluster (*K* 1 to 15) ± standard deviation. (**b**) ∆*K* for each value of *K*, calculated in Structure Harvester. STRUCTURE plots show the assignment probability of each greater glider (vertical tick marks) for *K* = 4 (**c**) and *K* = 2 (**d**). Geographic locations are listed below the graph and divided by the white-dashed lines.

Upon analysing the STRUCTURE results in Structure Harvester, the mean likelihood of each genetic cluster was observed to increase until *K* = 4 (Fig. 3a). Additionally, ∆*K* peaked at *K* = 4 before flattening out (Fig. 3b). Because of this, the most probably number of clusters was chosen to be *K* = 4, however *K* = 2 is also plotted since there was a slight peak in ∆*K* when *K* = 2 (Fig. 3d). The assignment results for the four clusters (Fig. 3c) showed cluster 3 was predominant in Gulguer, MSA, Escarpment N and Tallaganda, and cluster 4 was predominant in Meroo, Murramarang, Mogo E, Mogo W, Broulee, Eurobodalla and Monga. Cluster 2 was predominant in SMBNP. Escarpment S and Corramy had substantial admixture and did not appear to be dominated by a particular genetic cluster.

#### DAPC analysis

**Figure 4:**
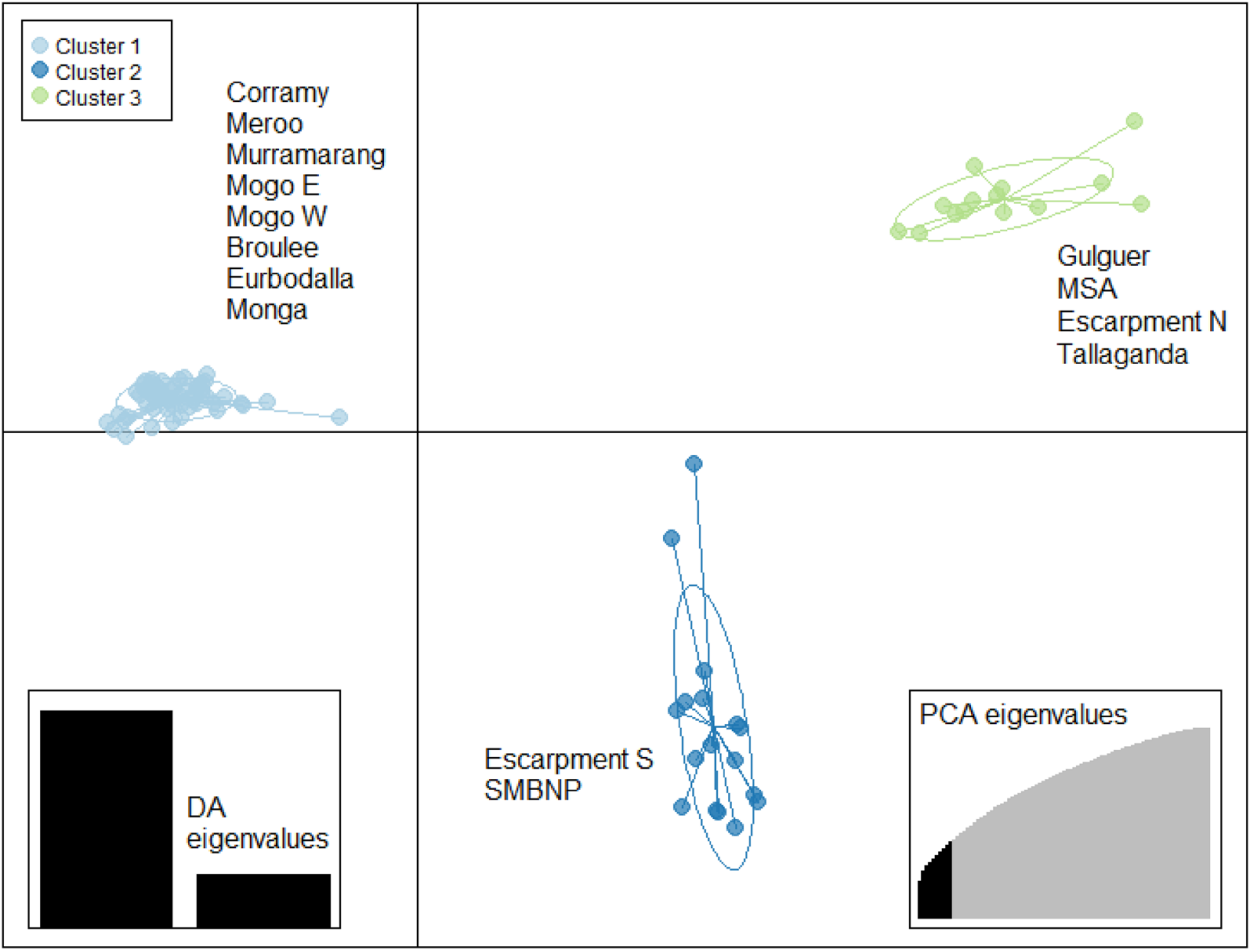
A Discriminant Analysis of Principal Components (DAPC) for 86 greater gliders in southern New South Wales, using 8493 SNPs. 10 PCA’s were retained. Dots represent individual greater gliders, and each colour represents a genetic cluster. Greater gliders were sampled from 14 geographic locations (labelled).

The DAPC analysis indicated that the 86 greater glider samples belonged to three genetic clusters (lowest BIC at *K* = 3) (Supplementary 3). The DAPC analysis assigned all greater gliders from Corramy, Meroo, Murramarang, Mogo E, Mogo W, Broulee, Eurobodalla and Monga to cluster one. Cluster two contained those from Escarpment S and SMBNP. The third and final cluster contained greater gliders from Gulguer, MSA, Escarpment N and Tallaganda (Fig. 4).

#### AMOVA results

The AMOVA supported the DAPC and STRUCTURE results, with significant genetic differentiation detected between greater glider sampling locations (21%). Most of the genetic variation was found to be within samples (76%), and only 3% of the genetic variation was apportioned between samples within sampling sites (Table 3). The de novo groups detected from the STRUCTURE analysis and DAPC analysis only slightly increased the percentage of variation detected between groups (Table 3).

**Table 3:** Greater glider Analysis of Molecular Variance (AMOVA) for the geographic sampling sites and the de novo groups generated from the STRUCTURE analysis (*K* = 4) and DAPC analysis (*K* = 3).

Analysis	Df	Sum of squares	Mean square	%	P-value
**1. Sampling locations**					
Between locations	13	60,291.02	4637.77	20.86	0.01
Between samples within locations	72	85,244.68	1183.95	3.37	0.05
Within samples	86	93,494.54	1087.15	75.77	0.01
Total	171	239,030.24	1397.838	100.00	
**2. STRUCTURE de novo groups**					
Between STRUCTURE groups	3	37,468.32	12,489.44	21.80	0.01
Between samples within STRUCTURE groups	82	108,067.38	1317.90	7.50	0.01
Within samples	86	93,494.54	1087.15	70.69	0.01
Total	171	239,030.24	1397.84	100.00	
**3. DAPC de novo groups**					
Between DAPC groups	2	34,545.57	17,272.79	22.54	0.01
Between samples within DAPC groups	83	110,990.13	1337.23	7.99	0.01
Within samples	86	93,494.54	1087.15	69.47	0.01
Total	171	239,030.24	1397.84	100.00	

*Df* degrees of freedom.

#### PCA results

**Figure 5:**
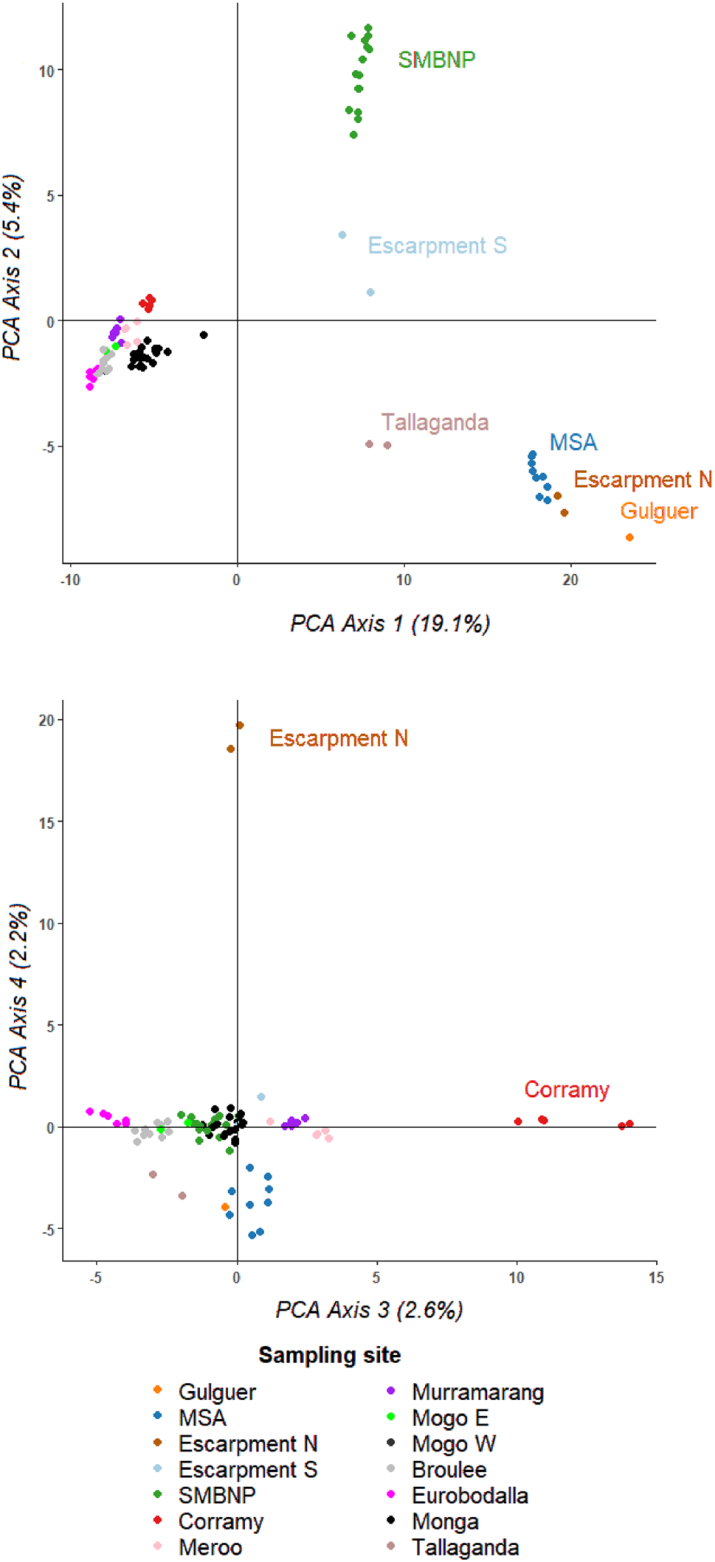
Pearson Principal Component analysis (PCA) of 86 greater gliders and 14 geographic locations, using 8493 genome-wide SNPs. Each dot represents an individual and the colours represent the sampling site (legend below the plots). Outgroups are named within the plots themselves. **Top:** The first two principal components accounted for 24.5% of the genetic variation. **Bottom:** The third and fourth PCA axes accounted for 4.8% of the genetic variation.

The Pearson Principal Component analysis (PCA) detected patterns of genetic structure amongst the 14 sampling sites. The first axis explained 19.1% of the genetic variation, and when the first four axes were combined, they cumulatively explained 29.3% of the genetic variation (Fig. 5). Axis 1 showed clear separation of Gulguer, MSA, Escarpment N, Tallaganda, Escarpment S and SMBNP greater gliders. Axis 2 showed the separation of SMBNP. Axis 3 showed the separation of Corramy, and Axis 4 showed the separation of Escarpment N greater gliders (Fig. 5).

### Isolation by distance (IBD) analysis

**Figure 6:**
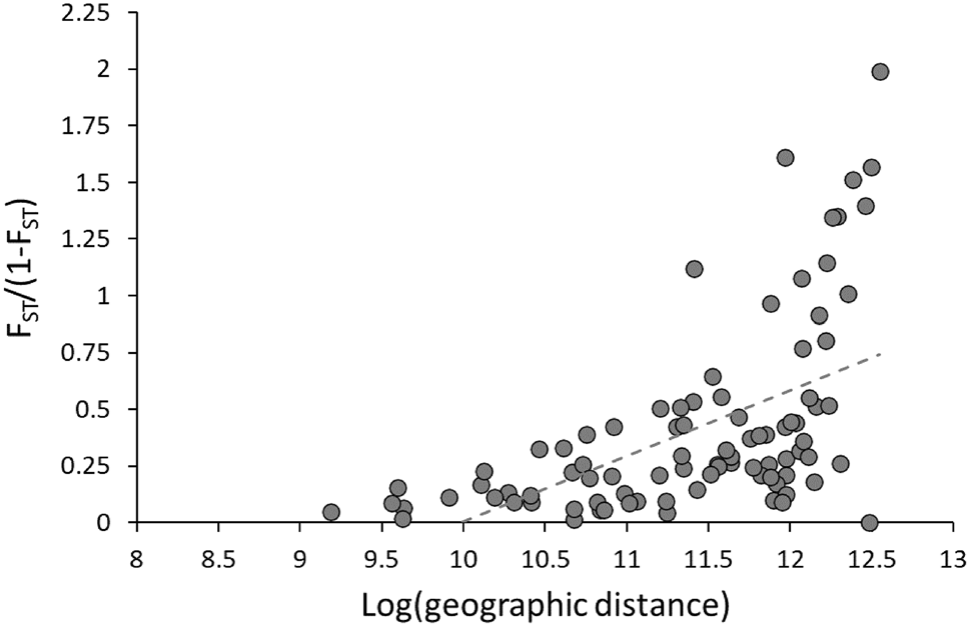
Isolation By Distance (IBD) analysis of southern NSW greater gliders using 8493 genome-wide SNPs and 14 geographic locations. Geographic distance had a significant effect on the genetic distances (F_ST_/1 − F_ST_) of greater gliders (Mantel statistic r = 0.6267, p < 0.01).

There was a significant IBD effect detected, with genetic distances strongly influenced by the geographic distances of greater gliders (Mantel r statistic = 0.627, p < 0.01) (Fig. 6).

### Effective population sizes of greater glider sampling sites

Greater glider effective population sizes (Ne) were calculated for locations where five or more individuals were sampled. Ne ranged from 6 (Eurobodalla) to 5058 (MSA), with an average Ne of 678 (± SD = 1771) (Table 4). Meroo, Murramarang and Eurobodalla all had small effective population sizes (Ne < 10). Monga (Ne = 161) and MSA (Ne = 5058) had the largest effective population sizes and were the only two locations where Ne was greater than 100 (Table 4).

**Table 4:** Contemporary effective population size (Ne) of greater gliders.

Sampling site	Ne	CI for Ne	
		Lower	Upper
MSA	5058.4	∞	∞
SMBNP	45.1	12.1	25.4
Corramy	51.0	9.1	∞
Meroo	8.2	2.9	∞
Murramarang	7.8	3.0	∞
Broulee	87.7	20.1	734.8
Eurobodalla	6.2	2.7	17.1
Monga	160.8	28.9	∞

Ne values were calculated for sites with 5 or more samples, using 8493 SNPs in NeEstimator 2.1. Upper and lower confidence intervals are displayed based on jackknife resampling. ∞ = infinity.

## Discussion

The increase in heatwaves and wildfires as predicted under a changing climate, significantly threatens the greater glider. Changes in the suitability of habitat can occur rapidly^38,40^, and have long lasting consequences on population size and thus ongoing genetic viability. Furthermore, because of these heat events, the probability for wildfires increases greatly and these stochastic events further impact the viability of populations. Our main findings identify a concerning pattern where all populations sampled, except one (MSA), had low effective population size and thus were at a heightened risk of local extinction. Additionally, we detected substantial genetic structure across NSW and a potential adaptation to temperature that may have implications for future management of greater gliders under a changing climate.

### Baseline genetic data following fire

There are few studies that have monitored changes to genetic diversity and effective population size of vertebrates after bushfires^95^, primarily due to a lack of baseline data. Catullo et al.^96^ discuss the importance of having pre-fire within-species diversity estimates for the effective monitoring of species recovery following the 2019–2020 Australian bushfires, however such data is not always available. Conservation genomic studies are crucial for well-informed genetic management decisions^16^. We demonstrate that baseline genetics are needed before these events happen as this allows for prioritization of population management and recovery actions, especially for endangered species where genetic declines are ongoing, and events induced by climate change only further exasperate these issues. Mcmahon et al.^51^ highlights the importance of genomic data in developing a ‘conservation prior’ with the aim of avoiding ‘emergency room conservation’. This involves identifying early on, viable populations with adaptive potential for prioritized preservation^51^.

Here we provide the first baseline genetic data for greater gliders across half of their distribution within New South Wales and summarize the conservation actions required for these locations (Supplementary 4). The locations used in this study have no prior genetic information available. Only one published study has examined greater glider genetics (using microsatellites) in NSW, and it was conducted over 20 years ago in Tumut, 130 km away from the nearest site (Tallaganda)^53^. Our analyses provide crucial baseline data for which fire-effected greater glider populations (Corramy, Meroo, Murramarang, Mogo E, Mogo W, Monga and Tallaganda) and endangered populations (SMBNP, Eurobodalla) can be monitored through time. Greater gliders are extremely susceptible to population decline and extinction, particularly in response to fire that partially or completely consumes the canopy^97^. Lindenmayer et al.^36^ reported low greater glider abundances in high severity burn sites in the Central Highlands of Victoria, and May-Stubbles et al.^37^ observed the same pattern within high severity burn sites of Monga National Park, NSW. This is due to the species’ specialist eucalypt diet and sensitivity to high temperatures^29–31,37,45,98^. Fire has also caused significant declines in the past, with greater gliders considered locally extinct after a fire burnt 90% of the Royal National Park (NSW) before they were rediscovered in 2012^99^. The species distribution continues to shrink, with greater gliders declared locally extinct in Booderee National Park (NSW) after the last sighting was reported in 2006^100^. Isolation is believed to be the main cause of this local extinction, having entered an extinction debt (a time lag between past events and their extinction level impact), which was likely then further exasperated by a wildfire that burnt 50% of the park in 2003^100^. Considering the negative effects of fire and isolation on greater glider population persistence, baseline genetic data is vital for species conservation by identifying priority sites and to inform genetic management actions.

### Genetic diversity

We obtained NSW genetic samples within 2 years of the 2019–2020 bushfires, with the goal of generating baseline genetic diversity estimates before significant generational genetic effects could be observed. Greater gliders at MSA displayed evidence of inbreeding (F_IS_ = 0.116), despite having high genetic diversity and a large contemporary effective population size. Parts of MSA experienced a wildfire in 2001^101^, which may have contributed to this. Escarpment N and MSA are linked by more than 100,000 ha of continuous habitat that is primarily a *Eucalyptus* and *Angophora* canopy (Woronora and Metropolitan Special Area)^101^. This further reinforces the importance of large continuous habitat in allowing populations to recover and remain resilient after stochastic events^7^. We propose that the greater glider population within MSA should be considered a stronghold for the species given its continuous connected habitat and large effective population size, however this location must be monitored over time to ensure inbreeding does not reduce genetic diversity. Efforts should be made to protect the area from wildfire events.

Greater gliders on the far south coast (Corramy, Mogo E, Mogo W, Broulee and Eurobodalla) displayed low genetic diversity (Ho = 0.090–0.105). These locations have experienced habitat loss and fragmentation from NSW state forestry logging (Mogo E, Mogo W) and isolation due to urban areas, farmland, lakes and rivers (Corramy, Broulee, Eurobodalla). The Eurobodalla population is especially isolated as they are surrounded by various water bodies, a highway and cleared land, and as a result the greater gliders in this location were categorized as part of an endangered population in NSW^57^. This is concerning as isolation was believed to be responsible for the extinction of greater gliders in Booderee National Park^100^, and Eurobodalla greater gliders displayed the lowest genetic diversity in our study area (Ho = 0.090). Since its listing as an endangered population by the NSW state government, no direct government intervention has been made for this population. Given its incredibly small effective population size, the Eurobodalla population is likely to go extinct. Despite being state-listed in the past as an endangered population,  this had no impact on direct conservation actions. Similarly, the population at Seven Mile Beach National Park (SMBNP), also previously state-listed as endangered, is highly isolated. However, the amount of available habitat appears to support relatively high numbers of gliders^102^ and moderate genetic diversity (Ho = 0.175). However, SMBNP may be a closed population (STRUCTURE and DAPC results both show that cluster two is predominantly SMBNP and Escarpment S), and thus vulnerable to future stochastic events. Direct action must be taken for SMBNP to avoid a repeat in genetic decline as observed in Eurobodalla. This baseline genetic data sets a pretext for future monitoring and informing management actions as they become required.

### Genetic structure and environmental adaptations

We expected to see genetic structure in the NSW greater gliders as their dispersal ability is dependent on trees and they have small home ranges of 1 to 3 hectares^28,103,104^. Substantial *Petauroides* genetic structure has been observed in other states, however the species delineation in NSW remains unclear^25^. As anticipated, high genetic structure was observed in the NSW greater gliders. Analyses showed significant genetic differentiation of Gulguer, MSA, Escarpment N and Tallaganda greater gliders to all other locations, and limited gene flow was evident through a significant IBD analysis^105^. Notably, the two greater gliders from Tallaganda were clustered with greater gliders 125 km away (DAPC cluster three) and were differentiated from those located only 30 km away in Monga National Park (DAPC cluster one, pairwise F_ST_ = 0.247). This could potentially be explained by the ~ 20 km stretch of cleared, agricultural land that has separated Tallaganda and Monga for over 200 years^106^.

The single greater glider from Gulguer was significantly differentiated from far south coast greater glider sampling locations, however these results should be considered cautiously as only one greater glider was sampled from Gulguer. Similarly, MSA and Escarpment N greater gliders were significantly differentiated from far south coast greater gliders. The limited gene flow and high genetic structure should be taken into consideration when developing management plans for the species following the 2019–2020 bushfires. Additionally, future research should combine our genetic data with existing data from Queensland and Victoria to further delineate the genetic structure of the three *Petauroides* species.

Greater glider populations should be managed separately where possible, particularly as we have shown that locations may be adapted to local climatic conditions such as temperature and precipitation. To avoid outbreeding depression associated with translocations, attention should be focused on conserving and connecting patches of fragmented forest (Supplementary 4). If translocations are required in the future, genetic data is essential, and individuals should only be selected if they have a similar genetic structure to the target population and are locally adapted to climatic conditions^107^. Here, we found maximum temperature of the warmest month may drive patterns of genetic variation in the greater glider genome. Gulguer, Escarpment N and SMBNP experienced the highest maximum temperature of the warmest month and mean temperature of the warmest quarter, while Eurobodalla and Broulee experienced the lowest. The isolated nature of the endangered Eurobodalla population is particularly concerning in the face of climate change, as remaining individuals may not be able to adapt fast enough and migration in/out of the area is currently not possible.

### Effective population size

 Ne is a criterion that is often used to support endangered species classifications  with the IUCN Red List of Threatened Species^108^ and aid in informing the prioritisation of conservation efforts Most studies suggest Ne ≥ 50 is needed to avoid inbreeding depression, while Ne ≥ 500 is needed to maintain evolutionary potential^23,24,109^. Frankham et al.^110^ challenge this notion and recommend these values be doubled. Only MSA greater gliders appeared to have minimum genetic requirements for their long-term persistence (Ne ≥ 500 Ne = 5058.4). The population at SMBNP, highly isolated with limited potential for immigration, does not meet these requirements (Ne = 45.1) and thus will be unlikely to persist long-term, without intervention. Other locations such as Meroo, Murramarang and Eurobodalla had extremely low effective population sizes (Ne < 10) and are subsequently at risk of inbreeding which is also reflected in the low observed heterozygosity values for these locations. Furthermore, low observed heterozygosity was observed in Mogo E, Mogo W and Broulee. Considering the low contemporary Ne values found in our study and the fact that greater gliders experienced an estimated 85% population loss as a result of the 2019–2020 catastrophic bushfires^2^, we support the up-listing of their conservation status across the entire state of NSW to endangered. Furthermore, greater gliders in unburnt, continuous forest habitat (MSA, Escarpment N) should be considered a critical stronghold that maintains a source population of greater gliders, contributing to the long-term population viability of gliders in the larger landscape. Further sampling in the Blue Mountains World Heritage Area, west of MSA, is also important given its size, protected status, and potential to support another large stronghold population.

### Management implications

We provide crucial baseline genetic data for the species prior to the genetic impacts from this natural disaster. Such information is essential to effectively monitor and inform species recovery^111,112^. We provide a detailed list of actions required for each location within our study (Supplementary 4), but also recognize that pragmatism is required when prioritizing expenditure of often limited funding available^51^. The identification of low genetic diversity and high genetic structure is concerning, and we strongly advise that future research directions should conduct similar genomic research at other locations across NSW to identify stronghold populations in areas of large, continuous forest. However, small and isolated coastal populations may present unique local adaptations that should also be preserved (e.g. SMBNP), as these could provide the adaptive potential needed to overcome the effects of climate change. In a post fire context, we exemplify the importance of collecting genetic data to elucidate ongoing issues and pre-emptively identify populations of greater extinction risk. Repeated wildfire events induced by a changing climate may continue to compound existing genetic effects, and long-term, self-sustainable solutions are sought to ensure species persistence into the future. Greater gliders have limited dispersal and are highly reliant on hollow-bearing trees. Thus restoring landscape connectivity (through creating and conserving wildlife corridors) and improving forest quality (by allowing disturbed, logged forests to recover and age to form hollows) is essential for the long-term persistence of this endangered species. Enhanced connectivity and improving habitat quality through increasing hollow availability, would not only promote gene flow and migration following natural disasters, but also improve the species’ ability to adapt and recover under a changing climate.

## Supplementary Information

Supplementary Information 1.

Supplementary Information 2.

Supplementary Information 3.

Supplementary Information 4.

## Data Availability

DArTseq data is available online through the figshare digital repository: 10.6084/m9.figshare.20235792.v1.
