# Supplementary material for: Conservation genomics of an endangered arboreal mammal following the 2019–2020 Australian megafire
Source: Sci Rep. 2023 Jan 10;13:480. doi: 10.1038/s41598-023-27587-3 (PMC9831986; doi:10.1038/s41598-023-27587-3)
Supplement: Supplementary file 1 — Supplementary Information 1. [file 41598_2023_27587_MOESM1_ESM.pdf]

Supplementary material 1. Table of genetic sample metadata.

Genetic sample label	Sample type	Date	Latitude	Longitude
GG-BWH-01	Donation	11-Jan-2020	-34.7420	150.6470
GG-MOGO-01	Donation	14-Aug-2021	-35.7969	150.0567
GG-SC-01	Donation	4-Mar-2021	-35.5299	150.1989
GG-D-04	Donation	3-Jun-2021	-35.3674	150.4339
GG-SM-05	Donation	30-Sep-2020	-34.6828	150.7630
GG-MTKEMBLA-04	Field capture	6-Apr-2021	-34.4640	150.7885
GG-KEMBLAGRANGE-20	Field capture	13-Apr-2021	-34.4643	150.7899
GG-7MBNP-22	Field capture	22-Jul-2020	-34.8055	150.7567
GG-7MBNP-03	Field capture	13-Aug-2020	-34.8018	150.7609
GG-GNR-03	Field capture	9-Nov-2020	-33.9361	150.6304
GG-7MBNP-05	Field capture	30-Nov-2020	-34.8203	150.7481
GG-7MBNP-30	Field capture	1-Dec-2020	-34.8154	150.7495
GG-7MBNP-12	Field capture	3-Dec-2020	-34.8202	150.7479
GG-7MBNP-11	Field capture	2-Dec-2020	-34.8200	150.7472
GG-7MBNP-06	Field capture	14-Feb-2021	-34.8211	150.7468
GG-7MBNP-20	Field capture	28-Apr-2021	-34.8000	150.7618
GG-7MBNP-10	Field capture	26-Apr-2021	-34.8003	150.7621
GG-7MBNP-70	Field capture	5-Jan-2021	-34.8124	150.7504
GG-7MBNP-23	Field capture	5-Feb-2021	-34.8121	150.7506
GG-7MBNP-07	Field capture	16-May-2021	-34.8031	150.7595
GG-7MBNP-01	Field capture	17-May-2021	-34.8010	150.7612
GG-7MBNP-20F	Field capture	18-May-2021	-34.8000	150.7629
GG-7MBNP-02	Field capture	19-May-2021	-34.8003	150.7621
GG-SW-10	Field capture	23-May-2021	-35.3581	149.6430
GG-SW-02	Field capture	23-May-2021	-35.3581	149.6430
GG-TR-02	Field capture	29-Aug-2021	-34.5081	150.5496
GG-TR-10	Field capture	29-Aug-2021	-34.5058	150.5512
GG-TR-01	Field capture	29-Aug-2021	-34.5058	150.5512
GG-TR-06	Field capture	30-Aug-2021	-34.5013	150.5602
GG-TR-40	Field capture	30-Aug-2021	-34.5038	150.5548
GG-TR-03	Field capture	30-Aug-2021	-34.5037	150.5548
GG-TR-04	Field capture	30-Aug-2021	-34.5039	150.5546
GG-TR-20	Field capture	31-Aug-2021	-34.4800	150.5742
GG-TR-21	Field capture	31-Aug-2021	-34.4741	150.5542
GG-BWH-20	Field capture	23-Sep-2021	-34.7393	150.6461
GG-CRP-02	Field capture	4-Oct-2021	-35.0976	150.5452
GG-CRP-04	Field capture	5-Oct-2021	-35.0987	150.5474
GG-CRP-60	Field capture	6-Oct-2021	-35.0976	150.5458
GG-CRP-70	Field capture	6-Oct-2021	-35.1001	150.5470
GG-CRP-03	Field capture	8-Oct-2021	-35.0968	150.5442
GG-MOGO-60	Field capture	11-Oct-2021	-35.7927	150.1669
GG-CONGO-02	Field capture	13-Oct-2021	-35.9718	150.1472
GG-CONGO-20	Field capture	13-Oct-2021	-35.9711	150.1482
GG-MUR-02	Field capture	14-Oct-2021	-35.6727	150.2467

GG-MONGA-02	Field capture	15-Oct-2021	-35.5619	149.9195
GG-MOGO-04	Field capture	18-Oct-2021	-35.7929	150.1680
GG-MOGO-08	Field capture	18-Oct-2021	-35.8145	150.0338
GG-PLUM-02	Field capture	19-Oct-2021	-35.5309	149.9336
GG-MUR-40	Field capture	20-Oct-2021	-35.6741	150.2470
GG-PLUM-07	Field capture	22-Oct-2021	-35.5299	149.9328
GG-PLUM-01	Field capture	22-Oct-2021	-35.5317	149.9340
GG-PLUM-40	Field capture	22-Oct-2021	-35.5328	149.9354
GG-PLUM-10	Field capture	22-Oct-2021	-35.5297	149.9332
GG-PLUM-20	Field capture	22-Oct-2021	-35.5297	149.9332
GG-MEROO-10	Field capture	23-Oct-2021	-35.4671	150.3760
GG-CONGO-40	Field capture	24-Oct-2021	-35.9695	150.1472
GG-MUR-04	Field capture	25-Oct-2021	-35.6789	150.2946
GG-MONGA-06	Field capture	27-Oct-2021	-35.5586	149.9210
GG-MONGA-04	Field capture	27-Oct-2021	-35.5386	149.9137
GG-PLUM-04	Field capture	30-Oct-2021	-35.5257	149.9421
GG-PLUM-70	Field capture	30-Oct-2021	-35.5258	149.9422
GG-PLUM-30	Field capture	31-Oct-2021	-35.5306	149.9343
GG-PLUM-03	Field capture	30-Oct-2021	-35.5256	149.9427
GG-MEROO-40	Field capture	1-Nov-2021	-35.4792	150.3891
GG-MEROO-20	Field capture	1-Nov-2021	-35.4772	150.3889
GG-MUR-01	Field capture	3-Nov-2021	-35.6784	150.2962
GG-MUR-07	Field capture	3-Nov-2021	-35.6784	150.2962
GG-CONGO-01	Field capture	5-Nov-2021	-35.9694	150.1486
GG-CONGO-10	Field capture	5-Nov-2021	-35.9696	150.1482
GG-MEROO-02	Field capture	6-Nov-2021	-35.4772	150.3904
GG-MUR-10	Field capture	7-Nov-2021	-35.6777	150.2934
GG-MEROO-01	Field capture	9-Nov-2021	-35.4785	150.3902
GG-CONGO-70	Field capture	11-Nov-2021	-35.9707	150.1480
GG-BRO-02	Field capture	11-Nov-2021	-35.8572	150.1634
GG-BRO-20	Field capture	13-Nov-2021	-35.8744	150.1495
GG-BRO-01	Field capture	13-Nov-2021	-35.8598	150.1638
GG-BRO-110	Field capture	14-Nov-2021	-35.8602	150.1601
GG-BRO-40	Field capture	13-Nov-2021	-35.8590	150.1618
GG-BRO-07	Field capture	14-Nov-2021	-35.8661	150.1512
GG-BRO-70	Field capture	14-Nov-2021	-35.8657	150.1512
GG-BRO-04	Field capture	14-Nov-2021	-35.8699	150.1505
GG-BRO-30	Field capture	15-Nov-2021	-35.8583	150.1619
GG-BRO-03	Field capture	16-Nov-2021	-35.8624	150.1532
GG-MONGA-01	Field capture	16-Nov-2021	-35.5613	149.9192
GG-MONGA-10	Field capture	17-Nov-2021	-35.5645	149.9185
GG-MONGA-70	Field capture	17-Nov-2021	-35.5672	149.9187
GG-MONGA-03	Field capture	17-Nov-2021	-35.5585	149.9219
GG-MONGA-40	Field capture	17-Nov-2021	-35.5629	149.9187
GG-MONGA-20	Field capture	17-Nov-2021	-35.5629	149.9187
GG-MONGA-06F	Field capture	17-Nov-2021	-35.5395	149.9176
