# Supplementary figures and images for: Conservation genomics of an endangered arboreal mammal following the 2019–2020 Australian megafire
Source: Sci Rep. 2023 Jan 10;13:480. doi: 10.1038/s41598-023-27587-3 (PMC9831986; doi:10.1038/s41598-023-27587-3)

**Supplementary 3.** BIC values for number of genetic clusters from DAPC analysis.

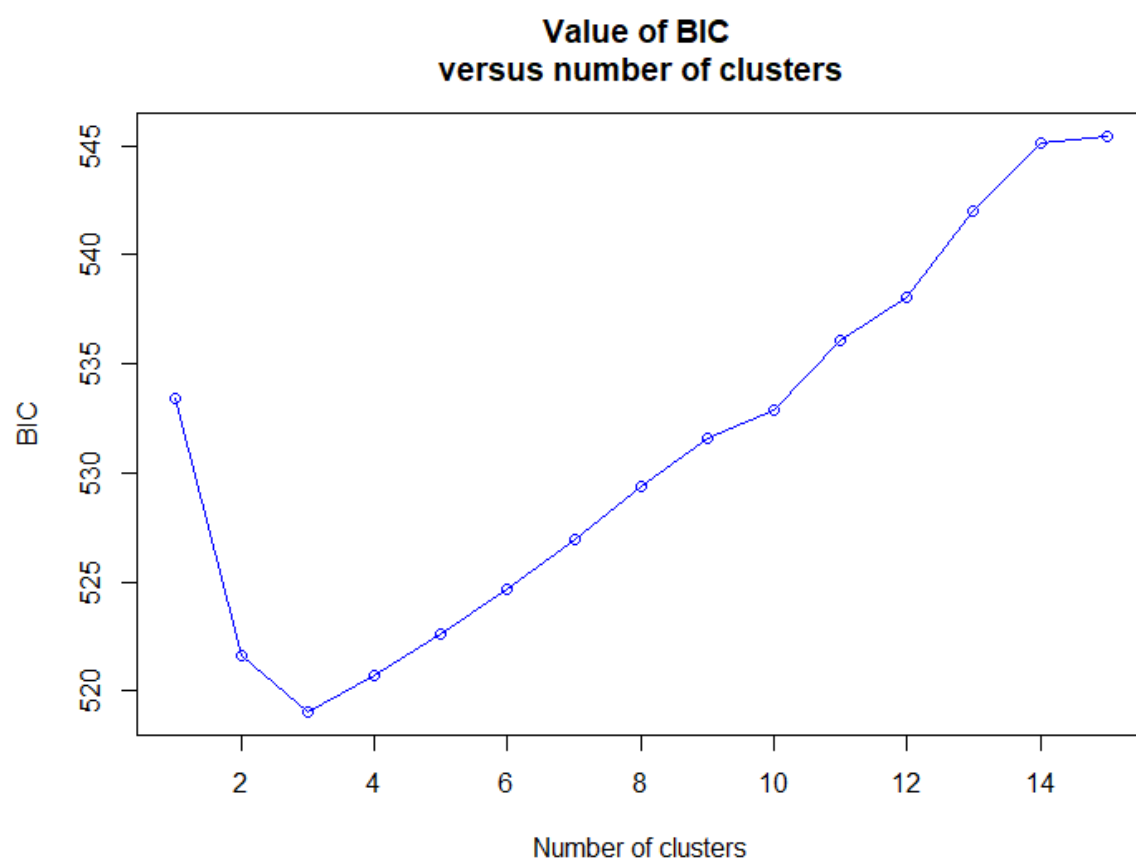

Supplement: Supplementary file 3 — Supplementary Information 3. [file 41598_2023_27587_MOESM3_ESM.pdf]
