# Supplementary material for: Conservation genomics of an endangered arboreal mammal following the 2019–2020 Australian megafire
Source: Sci Rep. 2023 Jan 10;13:480. doi: 10.1038/s41598-023-27587-3 (PMC9831986; doi:10.1038/s41598-023-27587-3)
Supplement: Supplementary file 4 — Supplementary Information 4. [file 41598_2023_27587_MOESM4_ESM.pdf]

Supplementary 4. Conservation actions for greater gliders at fourteen locations. Fire-affected refers to locations impacted by 2019/2020 megafire event (Y = Yes; N = No). * state-listed endangered population.

Region	Location	Fire affected?	Concerns	Conservation Actions
Cluster 3	Gulguer	N	Highly isolated, small population size with high potential for inbreeding (Ne < 10)	• Increase habitat connectivity and habitat quality (increase hollow availability) within the landscape as a top priority.<br>• Possible artificial augmentation or translocations (locally adapted individuals only).
	MSA	N	Inbreeding, experienced wildfire in 2001	• Protect as a stronghold population (higher genetic diversity than isolated areas, large effective population size).<br>• However, due to relatively high levels of inbreeding occurring further research is required (conduct further genomic research across the entire Metropolitan Special Area to create an effective management plan).
	Escarpment N	N	None	• It is connected via an existing vegetation corridor (the Illawarra Escarpment) to the MSA population.<br>• Conserve connectivity along the escarpment and continue to observe and monitor.
	Tallaganda	Y	Potential genetic bottleneck post-fire	• As it is part of a known, large population then further genetic research is required to determine if this is another stronghold population (sample size was small in this study).<br>• We propose a long-term landscape corridor connectivity project to reconnect Tallaganda and Monga National Park.
Cluster 2	Escarpment S	N	None	• Part of a larger continuous landscape, further genetic research required as sample size was small in this study.<br>• Potential source of gene flow to SMBNP.<br>• Conserve connectivity along the escarpment and continue to observe and monitor.

	SMBNP*	N	Highly isolated, small population size, high potential for inbreeding	• Increase habitat connectivity within the landscape as a top priority. If translocations required, then Escarpment S is a potential source population (locally adapted individuals only).<br>• Improve habitat quality (increase hollow availability) to increase total use of the national park and thus increase overall population size.
Cluster 1	Corramy	Y	Low genetic diversity, isolated, potential genetic bottleneck post-fire	• Maintain and improve habitat connectivity in the landscape<br>• Improve habitat quality (increase hollow availability) within the landscape
	Meroo	Y	Low genetic diversity, small effective population size (Ne < 10), potential genetic bottleneck post-fire	• Maintain and improve habitat connectivity in the landscape<br>• Improve habitat quality (increase hollow availability) within the landscape<br>• Monitor population regularly postfire<br>• Consider in future a possible artificial augmentation or translocations (locally adapted individuals only)
	Murramarang	Y	Low genetic diversity, small effective population size (Ne < 10), potential genetic bottleneck post-fire	• Maintain and improve habitat connectivity in the landscape<br>• Improve habitat quality (increase hollow availability) within the landscape<br>• Monitor population regularly postfire
	Mogo E	Y	Low genetic diversity, fragmentation and population loss from native forest logging, potential genetic bottleneck post-fire	• Protection of native forests from logging<br>• Improve habitat quality (increase hollow availability) within the landscape<br>• Monitor population regularly postfire
	Mogo W	Y	Low genetic diversity, fragmentation and population loss from native forest logging, potential genetic bottleneck post-fire	• Protection of native forests from logging<br>• Improve habitat quality (increase hollow availability) within the landscape<br>• Monitor population regularly postfire

	Broulee	N	Low genetic diversity	• Maintaining and restoring habitat to ensure ongoing connectivity with wider landscape
	Monga	Y	Potential genetic bottleneck post-fire	• Monitor population regularly postfire<br>• Propose a long-term landscape corridor connectivity project to reconnect Tallaganda and Monga National Park
	Eurobodalla	N	Highly isolated, small effective population size (Ne < 10), high potential for inbreeding	• Increase habitat connectivity and habitat restoration within the landscape as a top priority<br>• Improve habitat quality (increase hollow availability) within the landscape<br>• Potential need for translocation following habitat connectivity restoration
